# 

**Published:** 1896-12

**Authors:** 


**ESTABLISHED 1855.** **SUBSCRIPTION, $2.00.**

### ATLANTA MEDICAL AND SURGICAL JOURNAL.

VOLUME XIII. NEW SERIES. ATLANTA, GA., DECEMBER, 1896. No. 10.

